# Comparative effectiveness of asthma interventions within a practice based research network

**DOI:** 10.1186/1472-6963-11-188

**Published:** 2011-08-16

**Authors:** Hazel Tapp, Lisa Hebert, Michael Dulin

**Affiliations:** 1 Department of Family Medicine, Carolinas HealthCare System, 2001 Vail Avenue, Charlotte, NC 28207. USA; 2 Carolinas Physicians Network, Carolinas HealthCare System, PO Box 32861, Charlotte, NC 28232, USA

**Keywords:** asthma, comparative effectiveness research, shared decision making, integrated approach to care

## Abstract

**Background:**

Asthma is a chronic lung disease that affects more than 23 million people in the United States, including 7 million children. Asthma is a difficult to manage chronic condition associated with disparities in health outcomes, poor medical compliance, and high healthcare costs. The research network coordinating this project includes hospitals, urgent care centers, and outpatient clinics within Carolinas Healthcare System that share a common electronic medical record and billing system allowing for rapid collection of clinical and demographic data. This study investigates the impact of three interventions on clinical outcomes for patients with asthma. Interventions are: an integrated approach to care that incorporates asthma management based on the chronic care model; a shared decision making intervention for asthma patients in underserved or disadvantaged populations; and a school based care approach that examines the efficacy of school-based programs to impact asthma outcomes including effectiveness of linkages between schools and the healthcare providers.

**Methods/Design:**

This study will include 95 Practices, 171 schools, and over 30,000 asthmatic patients. Five groups (A-E) will be evaluated to determine the effectiveness of three interventions. Group A is the usual care control group without electronic medical record (EMR). Group B practices are a second control group that has an EMR with decision support, asthma action plans, and population reports at baseline. A time delay design during year one converts practices in Group B to group C after receiving the integrated approach to care intervention. Four practices within Group C will receive the shared decision making intervention (and become group D). Group E will receive a school based care intervention through case management within the schools. A centralized database will be created with the goal of facilitating comparative effectiveness research on asthma outcomes specifically for this study. Patient and community level analysis will include results from patient surveys, focus groups, and asthma patient density mapping. Community variables such as income and housing density will be mapped for comparison. Outcomes to be measured are reduced hospitalizations and emergency department visits; improved adherence to medication; improved quality of life; reduced school absenteeism; improved self-efficacy and improved school performance.

**Discussion:**

Identifying new mechanisms that improve the delivery of asthma care is an important step towards advancing patient outcomes, avoiding preventable Emergency Department visits and hospitalizations, while simultaneously reducing overall healthcare costs.

## Background

### Significance

Asthma is a chronic lung disease that affects more than 23 million people in the United States, including approximately 7 million children [1,2] The burden of asthma in the U.S. is high, accounting annually for 2 million emergency department visits, 504,000 hospitalizations, 13.6 million physician office visits, and over 4,200 deaths while resulting in $15 billion in direct medical costs [3-5]

In North Carolina during 2007, the lifetime prevalence of asthma in adults was 12.1%, impacting over 800,000 individuals [6] The prevalence of asthma was disproportionately higher in African American and Native American populations as well as low income populations [7] Asthma prevalence for the state also reached 17.8% in children under the age of 17 [7] Unfortunately, not only is asthma prevalence increasing in the Carolinas, but many patients suffering with asthma lack adequate control of their symptoms. Indeed, over 50% of adults noted asthma symptoms more than once per week and 21% had daily symptoms [7] This resulted in one-third of adults losing at least one day of work because of their asthma over the prior 12 months, and 24% of asthma patients having at least one Emergency Department (ED) or urgent care visit related to their asthma during this same period of time [7] Rates of ED utilization for asthma management were also almost 200% higher for minority children than non-minority children.

Our state's asthmatic patients also frequently lack a usual source of care, and 45% of asthmatics went without a visit to their regular physician over the past year [7] Patients with asthma also report a lower quality of life, with 18% of all asthmatic patients rating their health overall as poor [6] Hospitalization rates are also higher for asthmatic patients and a significant health expense for the state. Over $88 million was spent on hospitalizations for asthma in 2004 costing on average $8,259 per hospital stay.

The 2009 Institute of Medicine (IOM) report identified two priorities in the need for comparative effectiveness research on asthma including the need to study an integrated approach to care and shared decision making [8] The agency for healthcare quality and research (AHRQ) has also placed asthma on the priority list of conditions for comparative effectiveness research with particular interest in impacting populations that are low-income, minority groups, women, children, elderly individuals, and individuals with special health care needs, such as those who live in inner-city and rural areas. This attention to asthma is related to the burden of the disease on the U.S. population, disparities on outcomes for asthmatic patients, and the lack of knowledge of how to improve adherence to medications and subsequently improve asthma outcomes [9-17] Gaps in our knowledge of the optimal medical management and predictors of asthma outcomes (including environmental triggers) are also present and need to be addressed by comparative effectiveness studies [18-24].

Potential solutions include more comprehensive asthma management strategies that build upon existing successes in the development of integrated care systems; special emphasis on targeting high-risk populations; the use of self-management and shared decision making approaches to care; and school-based interventions that can be linked with primary care providers.

The National Asthma Education and Prevention Program (NAEPP) asthma guidelines emphasize that the goal for optimal asthma control is to reduce both impairment and risk, and recommend a stepwise approach to pharmacotherapy [25,26]. Measurement of asthma control can be complex, encompassing physical examination, objective tests, and patient history [26]. Well-controlled asthma is characterized by: experience of symptoms and use of a rescue medication twice a week or less, no early morning or nighttime awakenings, no limitations on activities of daily living, normal forced expiratory volume in 1 second (FEV_1_) or peak expiratory flow (PEF) test results, and controlled asthma as determined by physician and patient assessments [27].

Prevention and reduction of asthma exacerbations are key to reducing risk associated with asthma. NAEPP guidelines define asthma exacerbation as an "acute or sub-acute episode of progressively worsening shortness of breath, cough, wheezing, and chest tightness--or some combination of these symptoms" [25] Although most asthma exacerbations are treated in an outpatient setting, they present considerable difficulty for patients, including increased healthcare utilization, lost work productivity, school absences and increased healthcare costs [28].

One individualized behavioral approach to asthma disease management is the use of an asthma action plan, developed through asthma education activities to increase a patient's knowledge and skills with regard to asthma control [25,29-31] The National Institute of Allergy and Infectious Diseases (NIAID) inner-city asthma studies program found that a home-based approach tailored to an individual child's specific risk factors provided a more effective intervention strategy, especially with the inclusion of a written asthma action plan [32,33].

## Methods/Design

This study received ethics approval from the Institutional Review Board of Carolinas HealthCare System.

### Description of all interventions

**Figure 1:**
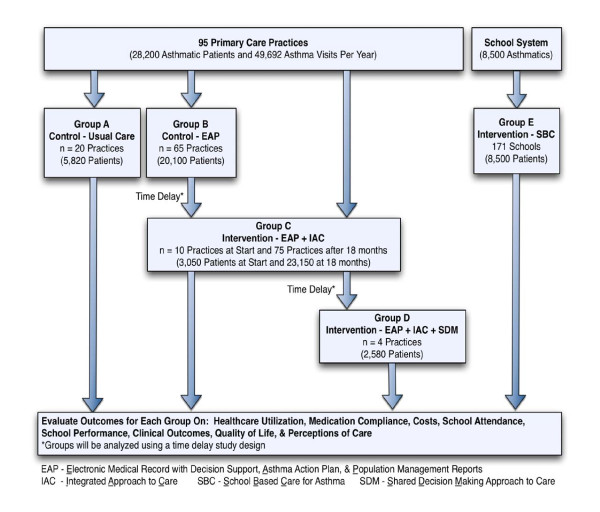
**Study Design**. This study will include 95 Practices, 171 schools, and over 30,700 asthmatic patients. Five groups (A-E) will be evaluated to determine the effectiveness of each intervention. Group A is the usual care control group. Group B practices will provide a second control group with an EMR with decision support, Asthma action plans, and Population reports (EAP) at baseline. A time delay design will be used as all practices in Group B will receive the Integrated Approach to Care (IAC) intervention with 10-12 practices receiving the intervention every 2 months over a 1 year period of time. Four practices within Group C will receive the Shared Decision Making (SDM) approach intervention with 1 practice receiving the intervention every 4 months over a 2 year period. Group E will receive School Based Care (SBC) through case management within the schools.

This study will include 95 Practices, 171 schools, and over 30,700 asthmatic patients. The interventions to be compared will include (Figure 1, Table 1): Group A: control practices providing usual care; Group B: control practices with a centralized electronic medical record (EMR), decision support tools, and population management tools; Group C: intervention practices have all the tools of Group B, but use an integrated system based on the Chronic Care Model to improve asthma outcomes; Group D: intervention practices will have all tools available to Group C with the addition of implementation of a shared decision making approach to care; and Group E: patients will receive additional care management through school-based nurses and care managers.

**Table 1:** Asthmatic Patients Seen in the CHS System 2008

	Number of Patients	Number of Clinic Visits
Total Number of Unique Patients with an Asthma Diagnosis	38,634	77,582

African American Race	14,168	34,551

Hispanic Ethnicity	2,043	4,596

Age < 18	11,058	21,357

Number within Mecklenburg County	16,458	41,961

Uninsured, Medicaid, or Medicare	13,564	31,022

Number of Hospitalizations for Asthma	10,321	NA

Number Emergency Room Visits	30,121	NA

CHS School Children with Asthma	8,500	NA

Three main interventions are planned:

#### 1) An integrated approach to care (IAC) for asthma management

Here our objective is to compare the effectiveness of an integrated approach to asthma management based on the chronic care model (CCM) with a non-integrated episodic care model (usual care control). This model based on the CCM, includes decision support, an electronic Asthma Action Plan, population management tools, training in the practice redesign and rapid cycle process improvement by a quality improvement coach, and linkages to community resources. This approach will be compared to control practices (A) and practices that receive the tools without training with a coach (B).

#### 2) A Shared Decision Making (SDM) approach

The objective is to compare the effectiveness of a combined shared decision making and IAC approach (C) with the integrated approach alone and with usual care (control). This model is based on the successful intervention developed by Wilson and colleagues that changes the dialog between patients and physicians to positively impact patient medication compliance and asthma self-management. This model will be implemented in select practices that have already implemented the IAC approach to determine if additional benefit can be gained using SDM. Practices receiving the SDM intervention also primarily serve disadvantaged populations including the uninsured, Medicaid patients, disabled Medicare patients, and minority groups.

#### 3) A School-Based Care (SBC) Approach

Here our objective is to compare the effectiveness of a school-based approach to care with the integrated approach to care and with and without the shared decision making approach to care and against the control. This intervention will provide an electronic data capture system to a robust CDC funded school-based intervention to assist with evaluation and to link the school-based care team with primary care providers. This system has identified almost 8,500 asthmatic children who will be a part of this intervention. Outcomes for children will be compared to children in control practices as well as practices in the IAC and SDM approach.

#### Clinical Outcomes

The major clinical outcomes goals are: reduced hospitalizations and emergency department visits; improved adherence to medication; improve quality of life; reduced school absenteeism; improved self-efficacy and improved school performance. Associated quality goals are to improve percentage of patients with persistent asthma who are prescribed a controller medication; and those with asthma who receive a flu vaccination. Quality goals will be measured by the percentage of patients who reach the goal out of all patients identified with the disease. The three interventions that will be undertaken each have the potential to impact these goals. The data collected for the evaluation of the three approaches to asthma management will also be leveraged to identify other important variables that impact asthma outcomes including: pharmaceutical management, co-morbidities (Tobacco Use/Exposure, GERD, Allergic Rhinitis, Obesity, Sleep Apnea), patient demographics (age, sex, race/ethnicity, insurance status), and community level variables (neighborhood quality of life, build environment and transportation elements, pollution sources, and housing density).

### Setting

**Figure 2:**
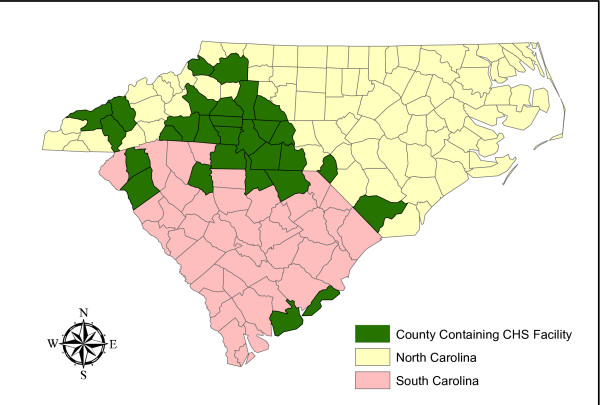
**Map of North and South Carolina Showing the Scope of Carolinas Healthcare System (CHS)**. Each county shaded in green is home to a CHS facility including the 32 hospitals and 95 clinics that will take part in this project.

The research network coordinating this project (The Mecklenburg Area Partnership for Primary Care Research, MAPPR) includes the hospitals, urgent care centers, and outpatient clinics within Carolinas Healthcare System (CHS) that share a common Electronic Medical Record (EMR) and billing system allowing for rapid collection of clinical and demographic data (Figure 2). This vertically integrated hospital system provides care to over 1.2 million patients including over 38,000 patients with a diagnosis of asthma. Between 2008 and 2009, these patients were responsible for almost 17,500 hospitalizations and 68,000 clinic visits. The research network includes a group of ambulatory clinics that together provide over 85% of care to the uninsured patients within the surrounding community. Inclusion of the 92,000 patients who receive care within these clinics allow the evaluation of the effectiveness of interventions for asthma management within a large population of poor and/or underserved community members. The MAPPR network includes the Mecklenburg County Health Department and the county school system (Charlotte Mecklenburg Schools, CMS). Indeed, CHS employs the 121 school nurses who assist with management of children with asthma in the school setting.

### Development of the Intervention

The approach is to implement the three separate but interrelated interventions across the community. The primary intervention, the integrated approach to asthma management, has been implemented in 10 practices, and through this project will be deployed in 65 additional practices. In addition, an active federally funded school-based system of care will be enhanced through this project allowing improved evaluation and comparison of outcomes between the school's system of care and other intervention as well as allowing the creation of a school to healthcare provider link.

#### Database Development

The overall strategy for this study is to create a centralized database for evaluation of comparative effectiveness of five different groups for management of asthma patients. The new database will draw information from the Carolinas Healthcare Systems billing and clinical databases, Medicaid claims data, school system data, chart abstraction, community level datasets, patient surveys and focus groups.

**Figure 3:**
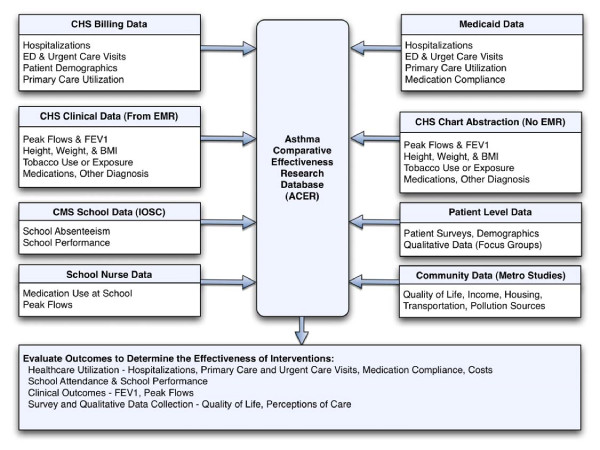
**Development of the Asthma Comparative Effectiveness Research Database (ACER)**. Data will be collected from 8 sources into the database including: Billing data, clinical data drawn electronically from the EMR, School data, Information collected by the school-based intervention, Medicaid data, clinic data via chart abstraction from control practices without an EMR, Patient level data from surveys and community-level data from the Center for Metropolitan Studies.

A centralized database will be created with the goal of facilitating comparative effectiveness research on asthma outcomes specifically for this study. The database named ACER (Asthma Comparative Effectiveness Research Database) will be setup using Microsoft SQL Server 2005 (Redmond, WA) and designed to support SAS (SAS Institute, Cary, NC) that is the standard analysis software used within the institution. The data will flow from 8 different sources including: the healthcare system's billing data, the healthcare system's clinical data, school data from the Institute for Social Capital, school nurse data, Medicaid claims data, patient level data (from surveys and focus groups), and community level data from the Center for Metropolitan Studies (Figure 3).

Any patient that has had a diagnosis of asthma recorded for billing purposes at any visit throughout the system since 2008 will be identified for the study as having asthma. This system recorded 38,634 unique patients for 2008 resulting in 77,582 visits to primary care practices. The unique medical records for each of these patients will be identified and the healthcare utilization patterns for these patients will be monitored prospectively through the course of the study. Additional data elements drawn from this dataset include patient demographics (age, gender, sex, race/ethnicity, and insurance type) as well as concurrent diagnoses.

For this study, clinical data from the EMR will be drawn from 75 of the study clinics (65 clinics in Group B and 10 clinics in Group C). All patients with asthma will be identified from billing data and the same unique medical record number will be used to pull these patient's clinical data. Data elements that can be pulled include: Tobacco Use/Exposure, assessment of daytime/nighttime symptoms, controller medication for persistent asthma, action plan given/updated and flu vaccine up to date.

The clinics in this study that are not connected to the EMR will be used as control practices. Clinical data from these clinics is abstracted from charts by the CHS quality team and will be ongoing during the 3 years of this study for comparison. Data collection will occur on a quarterly basis and will include: Tobacco Use/Exposure, assessment of daytime/nighttime symptoms, controller medication for persistent asthma, action plan given/updated, and flu vaccine up to date.

A local not for profit institute has been working with the Charlotte Mecklenburg School System for the past 4 years to store data for community-wide research. The Institute stores data on school absenteeism and school performance including End of Grade testing results. The school data will be added to the ACER server for the study to examine changes in school performance and absenteeism that are related to the study interventions.

The management of children with asthma within the school system through school-based nurses has enormous potential to improve asthma outcomes. This study will support the development of an electronic data capture system for school nurses. Data elements collected will include clinical measures including peak flows, asthma medications utilization, and the Asthma Action Plan.

The North Carolina Medicaid System is one of the most successful in the country in terms of providing high quality of care at a low cost [34] This system, Community Care Partners of North Carolina, works by incentivizing primary care physicians to provide preventative care through the assistance of case managers [35] CCPGM and Carolinas Healthcare System have a data sharing system allowing access to data for the Medicaid patients with asthma. The data are collected electronically and includes health services utilization (hospitalizations, ED and clinic visits) and data on medication compliance (prescriptions filled). These data are sent to CHS on a monthly basis and will continue to be shared for this study. Data for Medicaid will be matched based on patient name and birth date and entered into the ACER database for analysis.

### Patient Level Data

Patient level data will be collected using direct patient surveys, focus groups, and information exchange during the community forums.

#### Surveys

Depending on patient age, the patient survey will include the Mini Asthma Quality of Life Questionnaire (Mini-AQLQ) or Mini Pediatric Asthma Quality of Life Questionnaire (Mini-PAQLQ), the Asthma Therapy Assessment Questionnaire (ATAQ) and 2 additional five-point scale questions added by the research team. The Mini AQLQ (5 items) will licensed from QOLteck (West Sussex, UK) and used to collected data from all patients that are 17 years and older where this survey has been validated [36] The Mini PAQLQ will also be licensed from QOLteck, and this 13 question instrument will be used to assess quality of life in children between the ages of 7 and 17. The 5 point ATAQ questionnaire will be used to evaluate the effectiveness of asthma management for all patients. The pediatric version will be administered for children age 5-17 and adult version for patients age 17 and older [37]. The final questions are identical to those used by Wilson and colleagues to measure the patient's perceptions of a shared decision making approach and asthma self-efficacy [37-39] These questions will ask about the patients' perceptions of the quality of care they received and the influence they had versus the medical team in the development of their treatment plan. The finished survey will include 12 questions for adults and 20 questions for children under the age of 17 which will be printed on a single page for mailing.

Surveys will be mailed to a randomly selected group of asthma patients who receive care within the 95 clinics followed for this study every 6 months with the aim of collecting 100 surveys from each of the 5 groups at each time point. Patients will be incentivized to complete the survey with a $10 gift card for participation. Five of the clinics in this study are part of a network that provides care to disadvantaged patients including the uninsured, low-income minority populations, the indigent, disabled Medicare, and Medicaid patients. These populations tend to have frequent address changes and lower rates of literacy, resulting in lower rates of participation with mailed surveys. This group is of key importance for this research study and 4 of these 5 clinics have been targeted to receive additional interventions to improve asthma outcomes. Patients that complete the survey on-site will be provided with a $10 gift card to reimburse them for the time required to participate.

All survey data will be added to the ACER database on a weekly basis. Online survey results will be collected using Survey Monkey (http://Surveymonkey.com) and this data with the patient identifier and date of completion will be downloaded and transferred into ACER. Mailed surveys will be compiled by the research team and manually added to the database. In addition, the date that the survey was mailed and returned will be included for surveys returned by mail. If a survey is completed both online and via the mail, the mailed version will be discarded.

#### Focus Groups

Qualitative data will be collected through focus groups performed with patients (or their parents for children under age 17) and providers from each of the 5 groups throughout the study. Each focus group will have between 8-10 participants and will occur at baseline and every 6 months to collect qualitative information about the study. There will also be a focus group once every 1-3 months to evaluate the process and the monthly asthma meetings. There will be no recruitment for these, as the participants will be the regular attendees from the monthly asthma meetings (i.e. providers, key personnel from the clinic). A focus group guide for these sessions has been developed by the research team. Groups B-E will be asked to provide feedback about their perceptions of the study and its impact on their ability to receive or provide high quality asthma care. Particular interest will be focused in these groups on soliciting critical feedback about the project to be used for process improvement. Clinics in Group A will also be asked to participate, but these groups will be asked more general questions about their management of asthma patients and how they hope to improve care for asthma patients in the future. Focus group data will be analyzed and provided back to all participating practices and research team. Data from focus groups will be indentified only by practice and no individual data from the focus groups will be collected. Focus group data will not be added to the centralized ACER database.

#### Community Level Data

**Figure 4:**
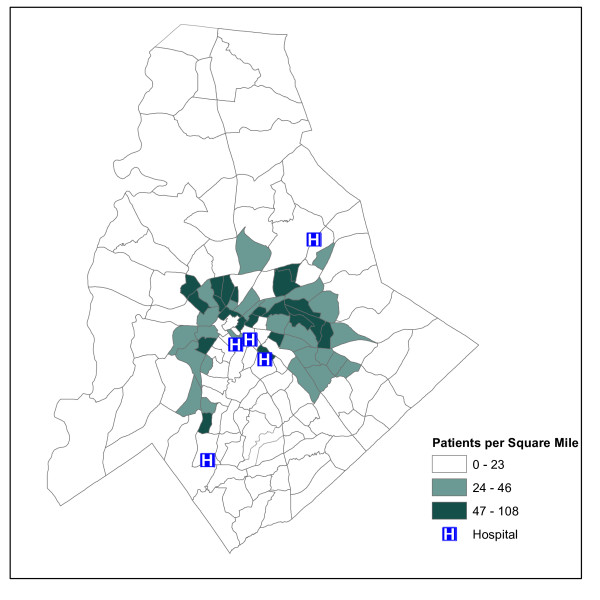
**Map of Asthma Diagnosis in Mecklenburg County**. Map of Mecklenburg County, NC showing Geographic Information Systems (GIS) analysis of the 6,800 patients with a primary diagnosis of asthma seen within the community clinic network over 1 year. The map shows patient density per square mile.

Through MAPPR's affiliation with the University of North Carolina at Charlotte, UNCC, and Center for Metropolitan Studies, geocoded data will be made available for this study showing: housing density, neighborhood quality of life, pollution sources, transportation and built environment elements, race/ethnicity, and household income. These data have been developed by the center or purchased from Claritas (New York, NY). We will geocode patient addresses for the ACER database which will allow the research team to examine and map asthma outcomes compared with other community level variables. One example of this technique can be seen in Figure 4, where patients with an asthma diagnosis within the clinic system for underserved or disadvantaged patients are mapped across Mecklenburg County. These maps can quickly be compared to other community-level data that will be shared by the Center for Metropolitan Studies.

### Analysis

#### Intervention Effectiveness Analysis

Comparisons between each group (A-E) and within Group E will be performed by statistical tests of regression model parameters. Specifically, we will generate a separate regression model for each of the six clinical measures and both quality measures (Table 2). Comparison groups will be defined as factors in the model. Propensity score methods will be used to control for differences in pharmaceutical management, patient demographics, co-morbidities, and community level variables between groups. Since Group E is not independent of Groups A-D, we will test for significant interactions between Group E and the other groups. A secondary analysis of the data will be performed to examine other important variables that impact asthma outcomes. This includes pharmaceutical management, co-morbidities (Tobacco Use/Exposure, GERD, Allergic Rhinitis, Obesity, Sleep Apnea), patient demographics (age, sex, race/ethnicity, insurance status), and community level variables (neighborhood quality of life, build environment and transportation elements, pollution sources, and housing density). In this analysis, we will build a regression model for each of the 6 clinical outcomes and use pharmaceutical management, patient demographics, co-morbidities, and community level variables as predictors. Regression tree methods will be used to help understand the potentially complicated interactions between these predictor variables. In both the primary and secondary analyses, linear mixed effects models will be used to account for practice-level effects within intervention group.

**Table 2:** Asthma Related Quality Improvement Goals and Outcome

Variable	Measure/Instrument	Data Source	Frequency
Quality of Life	Mini AQLQ, Mini PAQLQ, ATAQ	Individual Patient Surveys and Focus Groups	6 months

Hospitalization Rates	Inpatient Admission	CHS Hospital Data, Medicaid Data	Monthly

Acute Care Utilization	ED/Urgent Care Visits	CHS Data, Medicaid Data	Monthly

Primary Care Utilization	Primary Care Visits (Fam. Med, Peds, IM)	CHS Data, Medicaid Data	Monthly

School Attendance	Days Missed from School	School System (CMS) Data	6 months

School Performance	Test Scores	End of Grade (EOG) Test Scores	12 months

Demographics	Race/Ethnicity, Gender, Age, Insurance Type, Address	CHS System Data	Monthly

Income	Per capital household income, Home Value	Claritas, Census Data, UNC Urban Institute Data	6 Months

Housing Density/Location	Homes per sq. mile	Claritas, Census Data, UNC Urban Institute Data	6 Months

Other Medical Conditions	Obesity, Growth Delay, Developmental Delay, Upper Respirator Infections, Allergic Rhinitis, GERD, Diabetes, COPD, Cancer diagnosis, Hypertension, Congestive Heart Failure	CHS Hospital and clinical data	Monthly

Clinical Outcome Measures	FEV1, Peak Flow, BMI, Height, Tobacco Use, Tobacco Exposure, Vaccination Rates	CHS Electronic Medical Record/ Chart Abstraction	Monthly/ Quarterly

Medication Compliance	Asthma Medications Prescribed, Prescriptions Filled, Refill requests	CHS Electronic Prescribing Database, Medicaid Data	Monthly

#### Cost Analysis

The basic form of our analysis is that of a follow up study with three years of analysis. We will construct measures of service use and costs, by category of service as well as for all services combined, expressing all these measures in per-time-period terms for comparability across groups. This will permit comparison among groups to identify the effects of the intervention on health disparities, for example. Each of these dependent variables (use and cost, by service category) is specific to a calendar year. Thus the basic unit of analysis is the *person-year *of use or cost. We will use regression analysis to estimate the mean difference in outcome variables between groups, where the groups are identified by a dummy variable to examine effects of the integrated approach to asthma management based on the chronic care model (CCM), while holding constant other factors, and, in a more detailed analysis, by a set of dummy variables to examine the effects of the group interventions B through E, as compared with the control group A receiving usual care. Interactions between the dummy variable(s) and dummy variables indicating race/ethnicity will identify the effect of the CCM on health disparities. Cost savings to be achieved have the potential to be substantial, especially for the costs of hospitalization, which is a major cost driver. For example, using nationally representative data [40] we found that, among persons ages 19-64, the adjusted rate of preventable hospitalization for asthma for African American women was 2.2 times as great as that for non-Hispanic white women, while the comparable adjusted difference for men was 4.2 times as great. In this same age range, the adjusted rate for women was 2.2 times as great for Hispanics as for non-Hispanics, while among men the rate was 3.3 times as great for Hispanics.

Differences were much larger among those ages 65 and older, with rates 6 times higher than whites among men for African Americans, and over 8 times higher for Hispanics. Thus, even a modest reduction in the disparities of preventable hospitalization attributable to asthma affecting African Americans and Hispanics will substantially reduce total costs. Of course, in addition to reducing disparities in preventable hospitalization for African Americans and Hispanics, the intervention is likely to reduce preventable hospitalization related to asthma for all persons in the intervention groups.

## Discussion

Identifying new mechanisms that improve the delivery of asthma care is an important step towards advancing patient outcomes, avoiding preventable Emergency Department visits and hospitalizations, while simultaneously reducing overall healthcare costs. The wide range of patient types with asthma (eg. pediatrics vs. adults) as well as the varying degree of severity of the disease makes it difficult for a single approach to work universally. In addition, variation in primary care clinics and providers makes it challenging to implement new practices and concepts around asthma management such as shared decision making. To overcome these limitations, this study was designed with multiple interventions and two control groups. Perhaps most importantly, the shared decision making component of the intervention was designed to be developed and implemented using participatory methods to insure broad uptake and dissemination.

## Competing interests

The authors declare that they have no competing interests.

## Authors' contributions

All authors made significant contributions to the conception and design of this study and read and approved the final manuscript. HT and MD drafted the manuscript.

## Pre-publication history

The pre-publication history for this paper can be accessed here:

http://www.biomedcentral.com/1472-6963/11/188/prepub
